# Modeling leachate generation: practical scenarios for municipal solid waste landfills in Poland

**DOI:** 10.1007/s11356-022-23092-8

**Published:** 2022-09-21

**Authors:** Anna Podlasek

**Affiliations:** Institute of Civil Engineering, Warsaw University of Life Sciences (WULS-SGGW), Nowoursynowska 159 St, 02-776 Warsaw, Poland

**Keywords:** Leachate, Water balance, Sealing, Infiltration, HELP, UnSat Suite Plus

## Abstract

The idea of water balance calculations within the landfill is to determine the distribution of water input and output, and finally the volume of leachate generated. The scope of this data is essential for rational planning of water and wastewater management, and designing leachate drainage network and leachate treatment systems. The aim of this study was to assess the possible amounts of leachate generation regarding ten different scenarios of landfill sealing systems. The calculations were performed using the Hydrologic Evaluation of Landfill Performance (HELP) model. It was revealed that the greatest share among the components of water balance in the landfill has precipitation (on average 509 mm in the 5-year period of simulation), together with evapotranspiration (on average 391 mm in the 5-year period of simulation). The study shows that the minimum amount of leachate (797–803 m^3^/year) occurs when the best placement quality (=5) is regarded for the geomembrane installed in the bottom of the landfill. The maximum leachate generation (830 m^3^/year) was found for those scenarios in which only three layers of bottom sealing systems were adopted, with the worst placement quality (=1) assigned to geomembranes. The results of this study confirm that the application of multilayer sealing systems has visible impact on the reduction of leachate generation of around 33 m^3^/year.

## Introduction

The operation of municipal solid waste (MSW) landfills contributes to the emission of hazardous components that pose a serious threat to human health and the environment (Kumari et al. 2019; Vaverková et al. 2019). The type and intensity of nuisances depend to a large extent on internal factors related to the construction of the landfill, waste properties, or storage technology, as well as external factors related to the environment, including terrain topography or climate conditions (Safari and Baronian 2002; Agamuthu and Long 2007; Yang et al. 2015). The leachate, defined as infiltration water flowing through the landfill body, together with washed out water and dissolved waste components or products of biochemical reaction, constitutes a particular danger to the soil-water environment (Koda and Żakowicz 1998; Costa et al. 2019; Kotowska et al. 2020). It is also a danger to the components of the natural environment due to its toxicity (Vaverková et al. 2017; Przydatek 2019; Jabłońska-Trypuć et al. 2021). Leachate management is considered as one of the most important technological issues of landfill operation (Koda 2012); it is only proper management of leachate that can mitigate its negative impact on the environment. Suitable quantification of leachate generation is crucial for the needs of proper landfill design as a particular basis for the correct selection of parameters of drainage systems or retention reservoirs for temporary storage of the leachate (Yildiz et al. 2004). This is important in planning the amount of leachate that should be used during the recirculation (Abunama et al. 2017). In reference to the above, there is a real need for developing efficient methods of leachate purification and environment-friendly management (Talalaj 2015; Smol et al. 2017; Vaverková et al. 2018; Talalaj et al. 2019; Szymański et al. 2018). Also, the methods of evaluating the impact of leachate (Dąbrowska and Rykala 2021; Podlasek et al. 2021), as well as the methods of environmental risk assessment (Wowkonowicz et al. 2021), should be developed and improved.

In accordance with Polish standards (Regulation of the Minister of the Environment dated 30 April 2013 on landfills; Regulation of the Minister of Climate and the Environment dated 19 March 2021 amending the Regulation on landfills), hazardous waste landfills and non-hazardous waste landfills should be equipped with a leachate drainage system designed to ensure its reliable operation during the exploitation and for at least 30 years from its closure. The system of the landfill leachate collection and management usually consists of several elements, including a drainage net, trenches and reservoirs for leachate retention, components of the recirculation net, a leachate pumping station, discharge pipelines, and technical elements for distributing polluted waters on the surface of the landfill with the possible dilution of highly concentrated leachate for the needs of plant irrigation. The drainage system should be designed to meet hydraulic requirements throughout the entire period of biochemical activity of the landfill. Moreover, a well-designed drainage system is the key element for ensuring geotechnical safety because the location of the leachate depression curve within the landfill body has a significant impact on the global stability of the landfill slopes (Adamcova 2019; Koda et al. 2020). A poorly functioning leachate discharge system can lead to leachate effluence through the landfill slopes, resulting in contamination of the soil and water environment in adjacent areas (Rowe 2011; Koda et al. 2013).

The amount and composition of the leachate, as well as the velocity of its movement within the landfill body, can vary greatly (Wdowczyk and Szymańska-Pulikowska 2019). It depends mainly on the age of the landfill, the degree of waste compaction, and the techniques used during the deposition and forming of the landfill body. Długosz (2012) reported that the amount of leachate generated is impacted also by the terrain conditions, stage of the landfill operation, applied liners, soil features, and the type of vegetation covering the landfill. Komilis and Athiniotou (2014) revealed that a major source of leachate is the moisture of the over-saturated wastes, because monthly leachate amounts were much greater than the monthly precipitation volume infiltrating into the landfill. In semi-arid regions like Sousse in Tunisia, the major sources of leachate are not runoff and precipitation. The studies show that leachate generation is linked to biological activities and influenced by the type, amount, moisture, and compaction of wastes. Evapotranspiration is also an additional essential contributor to leachate generation (Frikha et al. 2017).

Abunama et al. (2017) reported that for the Palestine site conditions, almost 54% of the leachate originates from the initial moisture content of the stored wastes, whereas 31.7% and 14.3% are related to the infiltration and recirculation, respectively. Klimek et al. (2010) indicated that the application of the surface capping system is particularly important for the amount of leachate generated. Those authors revealed that for the annual precipitation of 500 mm, the amount of leachate generated in a landfill without surface sealing is four times greater than in a landfill where surface sealing was applied. In the absence of sufficient sealing of landfill cells, leachate can infiltrate through the soil profile and consequently reach the aquifer where it can freely migrate over considerable distances from the landfill. Therefore, from an engineering point of view, it is important to correctly design drainage systems that take the leachate out of the landfill cells into leachate tanks.

Engineering considerations related to drainage designing are primarily focused on the analytical calculations of water balance. Water balance is affected by several factors, variable during the operation of the landfill and after its closure, related to the technological aspects of landfilling and meteorological parameters, i.e., precipitation, temperature, speed and direction of wind, evapotranspiration, air humidity, inflow of water from external sources, and the properties of landfilled wastes (Koda and Żakowicz 1998). When calculating the water balance at landfill sites, it is usually assumed that 50–60% of rainfall infiltrates through poorly compacted wastes, whereas in well-compacted landfills the range of rainfall infiltration is up to 25%. For comparison, Machajski and Olearczyk (2008) reported that the average amounts of leachate generated in the landfill in relation to the average annual precipitation are 15–25% of the precipitation for heavily compacted wastes with the use of a compactor, and 25–50% of the precipitation for averagely compacted wastes by a dozer.

The simplest approach of estimating leachate generation is using the method in which the only factors taken into consideration are the annual rainfall distribution and the surface area of the landfill (Ibrahim et al. 2017). Yang et al. (2015) pointed out that considering only precipitation data in practical calculations leads to the underestimation of real leachate generation, especially when the moisture of wastes is relatively high. An inappropriate estimation of leachate generation results in an increased water head above the liner system, due to insufficient design capacities of the leachate collection system. Most regulations specify a maximum leachate head as 0.3 m, but a practical example shows that in Chinese conditions the leachate head can be even higher than 10 m (Shu et al. 2019). Subsequently, the high water level in a landfill body may lead potentially to leaching into the surrounding area and cause landfill instability.

Despite the widespread use of numerical modeling techniques worldwide, there are still not enough scientific studies presenting the possibilities of numerical modeling application for the purpose of leachate generation assessments in the Polish site conditions. Therefore, from the scientific point of view, a novelty of the presented approach is to fill this gap of knowledge and indicate whether it would be reliable to predict leachate generation from the landfills located in central Poland using the HELP model. Another important issue raised in the performed analysis is the placement quality of the introduced sealing materials, which could affect the intensity of leachate percolation through the layers of the capping system. The research brings also new insights to the current state of knowledge and could be of a considerable importance as it is focused on the assessment of leachate quantity instead of leachate quality which was more frequently analyzed in the scientific studies. This approach may be considered innovative as so far, the authors have not specified the exact amounts of leachate that can be produced in specific site conditions, instead of that they have rather focused on leachate quality and its impact on the quality of the environment. In reference to this, the main objective of this study was to compute the possible quantity of leachate generated in a model landfill following several scenarios of landfill bottom sealing and cover systems.

## Materials and methods

### Water balance method

**Fig. 1:**
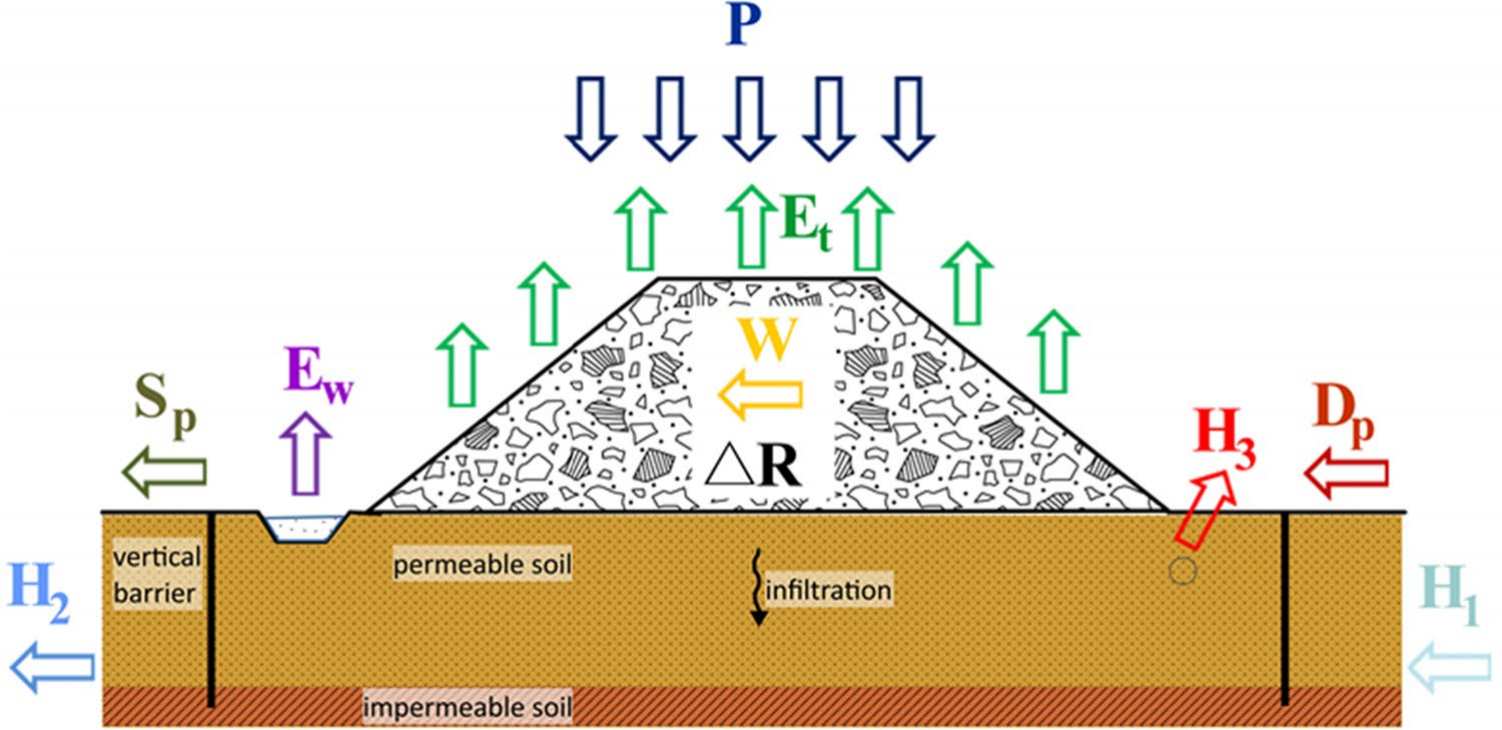
Components of water balance within the landfill site

The assumption that all water-infiltrating wastes become leachate is the basic approach for the water balance method (Fenn et al. 1975). The water balance (Fig. 1) can be calculated using several equations.

One of the most frequently equations reported in the literature can be expressed as follows:

1 $$P+W+{H}_1+{D}_p={E}_t+{E}_w+\Delta R+{S}_p+{H}_2+{H}_3$$

where

*P* is the precipitation, *W* is the water content in landfilled wastes, *H*_*1*_ is the underground water inflow, *D*_*p*_ is the surface water inflow, *E*_*t*_ is the evapotranspiration from the surface and landfill slopes, *E*_*w*_ is the evaporation from the retention reservoirs, Δ*R* is the effective capabilities of water retention by a landfill body, *S*_*p*_ is the surface outflow, *H*_*2*_ is the underground water outflow, and *H*_*3*_ is the leachate outflow collected by the drainage net.

In landfills isolated with vertical barriers, the water balance can be calculated using the equation:

2 $$P+W={E}_t+{E}_w+\Delta R+{H}_3$$

In computational practice, especially when designing drainage systems, observations and experiments on existing facilities are commonly used; hence, the equations for water balance calculations are often shortened to

3 $$L=P-{S}_p-{E}_t$$

where

*L* is the leachate, *P* is the precipitation, *S*_*p*_ is the surface outflow, and *E*_*t*_ is the evapotranspiration.

Leachate generation can be also effectively predicted using numerical modeling techniques (Grugnaletti et al. 2016). As reported by Min et al. (2010), numerical modeling is especially efficient when determining parameters of engineering liners, leachate collection systems, surface slope, and subsurface drainage. The most commonly used models in the hydrological evaluation of landfills are UNSAT-H (Fayer 2000), HYDRUS-1D (Šimůnek et al. 2005), and HELP (Schroeder et al. 1994). The HELP model was successively applied in engineering practice on landfills in the USA and is more frequently used for the assessment of leachate generation in different landfill sites in the world (Beck-Broichsitter et al. 2018).

### HELP model — a theoretical background

HELP is a deterministic model that allows for creating simulations of water flow through landfill layers, both in the case of operating and closed landfills. HELP incorporates modeling of vertical flow with regards to evaporation and infiltration and lateral flow, including runoff and lateral drainage (Mesania and Jennings 1998). The HELP model applies a discrete method of solution, concerning the calculation of the water balance layer by layer. HELP investigates the processes within the landfill, including the flow through the capping system and the waste body as well as the percolation through the bottom liners. The HELP model establishes up to four types of layers, representative for the landfill construction (Table 1).

**Table 1:** Layers adopted in the HELP model

No.	Layer	Function	Description
1	Vertical percolation layer	To support vegetation and moisture storage	The flow through this layer is based on the vertical movement of water downward due to gravity or upward due to evapotranspiration.
2	Barrier soil liner	To limit vertical drainage	The material of the layer is characterized by very low hydraulic conductivity.
3	Geomembrane liner	To control liquid drainage through the landfill profile	Designed to be practically impermeable. It is assumed that liquids can leak through geomembranes due to vapor diffusion through the intact geomembrane, leakage through manufacturing defects (pinholes), or leakage through construction defects in seams.
4	Lateral drainage layer	To promote lateral drainage	The layer is located above the liners. Typically consists of sandy-gravel or geosynthetic materials.

### Input data

**Fig. 2:**
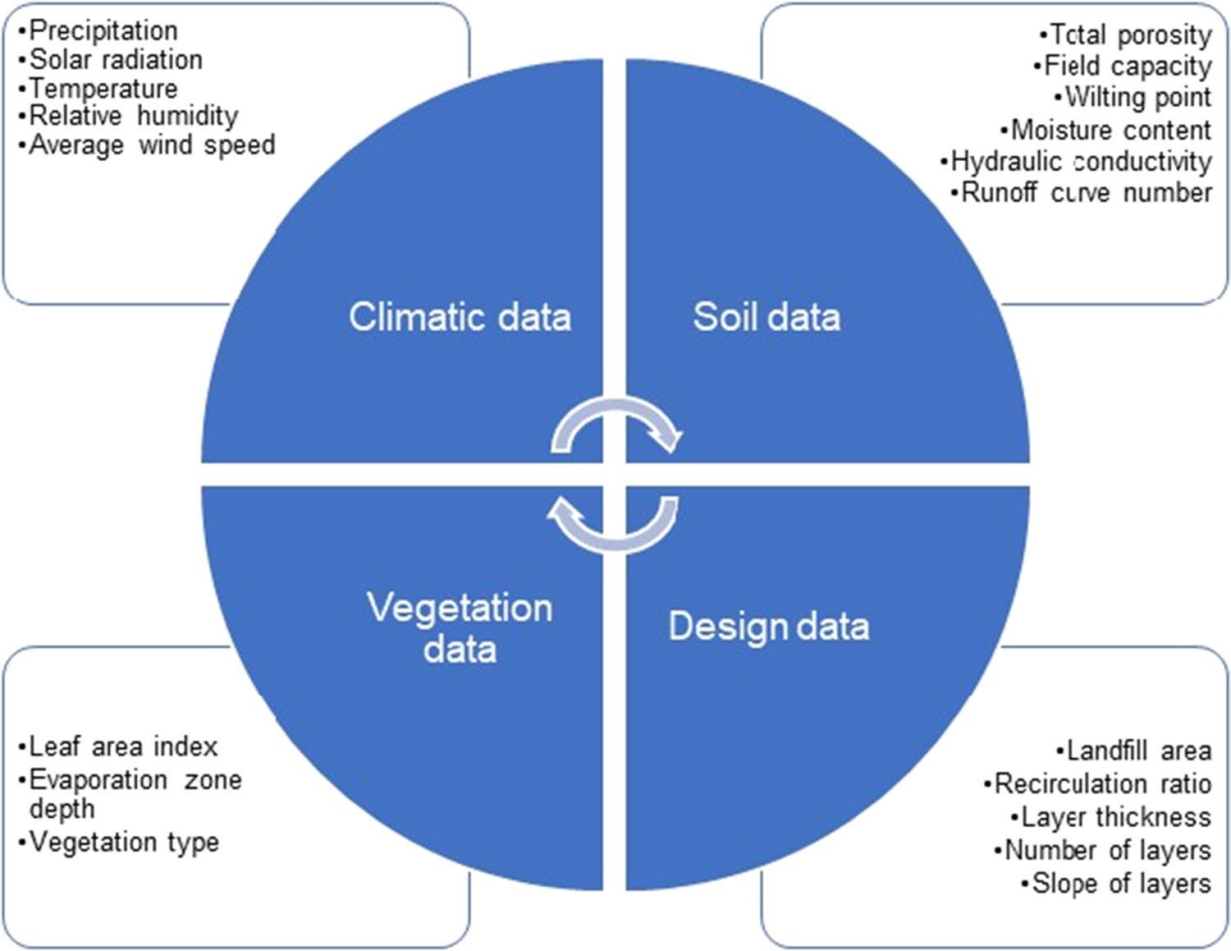
Set of parameters applied for modeling leachate generation in HELP

The HELP model requires data on climate, soil, vegetation, and design specifying the type and arrangement of the layers (Fig. 2). The weather data includes precipitation, temperature, and solar radiation. The key soil input data are porosity, field capacity, wilting point, and hydraulic conductivity (Table 2). The weather data were set using the weather generator incorporated in the HELP software and the monitoring results obtained from the local meteorological station.

**Table 2:** Parameters of materials adopted in the HELP model

Material	Category	Total porosity (vol/vol)	Field capacity (vol/vol)	Wilting point (vol/vol)	Saturated hydraulic conductivity (m/s)	Subsurface inflow (m/s)
Fine sandy loam	Vertical percolation layer	0.4730	0.2220	0.1040	5.2 × 10^−6^	0.0000
Coarse sand	Lateral drainage layer	0.4170	0.0450	0.0180	1.0 × 10^−4^	0.0000
Silty clay	Barrier soil liner	0.4790	0.3710	0.2510	2.5 × 10^−7^	0.0000
MSW	Vertical percolation layer	0.6710	0.2920	0.0770	1.0 × 10^−5^	0.0000
Sand	Lateral drainage layer	0.4370	0.0620	0.0240	5.8 × 10^−5^	0.0000
Fine sand	Lateral drainage layer	0.4570	0.0830	0.0330	3.1 × 10^−5^	0.0000
Sandy clay loam	Barrier soil liner	0.3980	0.2440	0.1360	1.2 × 10^−6^	0.0000
Drainage net	Geotextile and geonets	0.8500	0.0100	0.0050	1.0 × 10^−1^	0.0000

### Processes

In this research, the flow of the water into, through, and out of a landfill (with regard to the processes taking place on the surface and below the surface) was considered. All of the processes modeled in the HELP model were in accordance with the mathematical expressions presented by Bauerle (2016). In the performed analysis, the following processes were included:

1. Surface processes: snowmelt, interception or rainfall by vegetation, surface runoff, and evaporation.
2. Subsurface processes: soil-water evaporation, plant transpiration, vertical drainage, liner leakage, and lateral drainage.

In initial calculations, the surface processes were considered in the first stage and then, any water remaining was considered to infiltrate into the landfill body. The runoff was calculated on the basis of precipitation, land use, soil type, and moisture content. The infiltration was considered as the difference between the amount of precipitation, the sum of runoff, surface storage, and surface evaporation. The subsurface processes were taken into account once water had infiltrated into the landfill. Evapotranspiration was regarded as occurring up to the evaporative zone depth, and included the sum of soil-water evaporation and plant transpiration. Evapotranspiration was computed using a modified Penman method (Ritchie 1972). The range of plant transpiration was calculated with regard to the seasonal variation of the leaf area index (LAI). The vertical flow, forced by gravity, was modeled by a water storage and routing approach, which was considered for each segment of the model, where the water flows downward from the top to the bottom of a given segment. In the calculations, the following were included: hydrological properties of each segment, water storage capacity, and infiltration. Leachate recirculation was not modeled. Percolation through the soil barrier was treated as a vertical flow, following Darcy’s law. In the calculations, it was assumed that the barrier soil liners are saturated at all times and leak only when a positive head on the top surface of the liner exists. Thus, it was possible to model that any water moving into a liner can percolate through the liner. For computational purposes, the soil profile was partitioned into subprofiles. The first subprofile was set from the landfill surface to the bottom of the soil layer in the cover system. The second subprofile was regarded from the top of the waste layer to the base of the geomembrane liner system. The third subprofile was set below the second subprofile, to the base of the barrier soil layer in the bottom sealing system. Percolation through the geomembrane was considered as the flow through pinhole defects or as vapor diffusion. Leachate outflow was modeled as lateral drainage assuming a saturated flow.

### Modeling scenarios

The input data, including evapotranspiration, precipitation, temperature, and solar radiation, were set specifically for the considered location in central Poland. The evaporative zone depth was set as the average of the minimum and maximum evaporative zone depths for the selected site location. The leaf area index (LAI) was assigned according to the vegetation stage. In the calculations of leachate generation, an important factor was the saturated hydraulic conductivity (*k*_sat_) of wastes. The literature findings showed that the permeability of wastes can vary depending on different factors (Reddy et al. 2009), in the range of several orders of magnitude (Table 3).

**Table 3:** Values of *k*_sat_ of wastes

Source	Fleming (2011)	Fellner (2004)	Machado et al. (2010)	Jang et al. (2002)	This study
*k*_sat_ (m/s)	7 × 10^−6^	1–5 × 10^−9^	1 × 10^−5^–1 × 10^−8^	1.07 × 10^−5^–2.9 × 10^−6^	1 × 10^−5^

Several scenarios of MSW landfill construction were considered in calculations of leachate generation (Table 4). In the selected scenarios (1–7), the landfill consisted of 10 layers and a good stand of grass was considered for the vegetation. The geosynthetic layers applied in the bottom part of the landfill had variable placement quality (from 1 to 5). In scenarios 8–10, the landfill consisted of 7 layers. The arrangement of the geosynthetic layers in the bottom part was analogous to scenarios 1–7. The parameters of geomembranes regarded in the selected scenarios are summarized in Table 5.

**Table 4:** The concept of landfill layers considered in calculations for scenarios of leachate generation

Section of the landfill	Scenario 1	Scenario 2	Scenario 3	Scenario 4	Scenario 5	Scenario 6	Scenario 7	Scenario 8	Scenario 9	Scenario 10
Cover layers	Fine sandy loam	Fine sandy loam	Fine sandy loam	Fine sandy loam	Fine sandy loam	Fine sandy loam	Fine sandy loam	Fine sandy loam	Fine sandy loam	Fine sandy loam
	Coarse sand	Coarse sand	Coarse sand	Coarse sand	Coarse sand	Coarse sand	Coarse sand	Coarse sand	Coarse sand	Coarse sand
	Silty clay	Silty clay	Silty clay	Silty clay	Silty clay	Silty clay	Silty clay	Silty clay	Silty clay	Silty clay
Wastes	Municipal wastes	Municipal wastes	Municipal wastes	Municipal wastes	Municipal wastes	Municipal wastes	Municipal wastes	Municipal wastes	Municipal wastes	Municipal wastes
Bottom layers	Sand	Sand	Sand	Sand	Sand	Sand	Sand	Sand	Sand	Sand
	Drainage net	Drainage net	Drainage net	Drainage net	Drainage net	Drainage net	Drainage net	-	-	-
	HDPE	LDPE	PVC	BR	CPE	CSPE	EPDM	HDPE	LDPE	PVC
	Fine sand	Fine sand	Fine sand	Fine sand	Fine sand	Fine sand	Fine sand	-	-	-
	BR	BR	BR	BR	BR	BR	BR	-	-	-
	Sandy clay loam	Sandy clay loam	Sandy clay loam	Sandy clay loam	Sandy clay loam	Sandy clay loam	Sandy clay loam	Sandy clay loam	Sandy clay loam	Sandy clay loam

HDPE – high-density polyethylene, LDPE – low-density polyethylene, PVC – polyvinyl chloride, CPE – chlorinated polyethylene, CSPE – chlorosulfonated polyethylene, EPDM – ethylene propylene diene monomer, BR – butyl rubber

**Table 5:** Parameters of materials acting as geomembrane liners in the analyzed scenarios of leachate generation

Material	Saturated hydraulic conductivity (m/s)	Pinhole density (number of holes/ha)	Installation defects (number of holes/ha)	Placement quality (-)	Transmissivity (m^2^/s)
HDPE	2 × 10^−15^	2	2	1–5	0
BR	1 × 10^−14^	2	4	1–5	0
LDPE	4 × 10^−15^	2	4	1–5	0
PVC	4 × 10^−13^	2	4	1–5	0
CPE	4 × 10^−14^	2	4	1–5	0
CSPE	3 × 10^−14^	2	4	1–5	0
EPDM	2 × 10^−14^	2	4	1–5	0

### Statistical analysis

The results of calculations performed in the HELP model were subjected to statistical analysis using Statistica 12 software (StatSoft Inc., Tulsa, OK, USA). Box and whisker plots were prepared to show the range of mean values together with the standard error (SE) and standard deviation (SD) of the leachate generated in each scenario. A correlation matrix was prepared to indicate the relationships between the components of water balance in the landfill. The interpretation of the results was based on the guidelines reported by Rabiej (2018). The strength of the obtained correlation was analyzed after Hinkle et al. (2003).

## Results and discussion

**Fig. 3:**
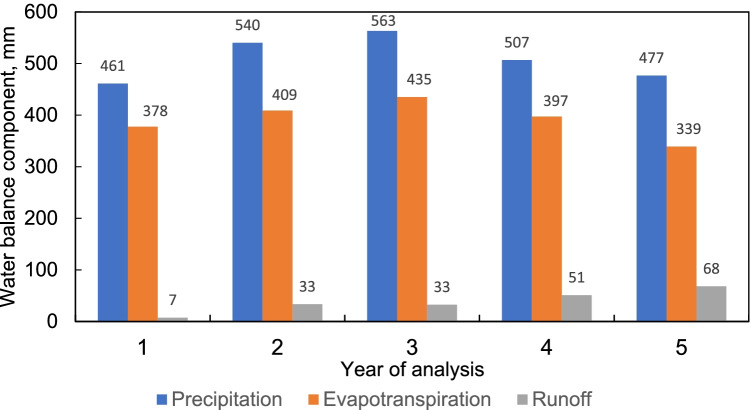
The distribution of water balance components in 5-year period of analysis

In the calculations performed in this study, it was found that the greatest share in the components of water balance in the landfill has precipitation (Fig. 3).

**Fig. 4:**
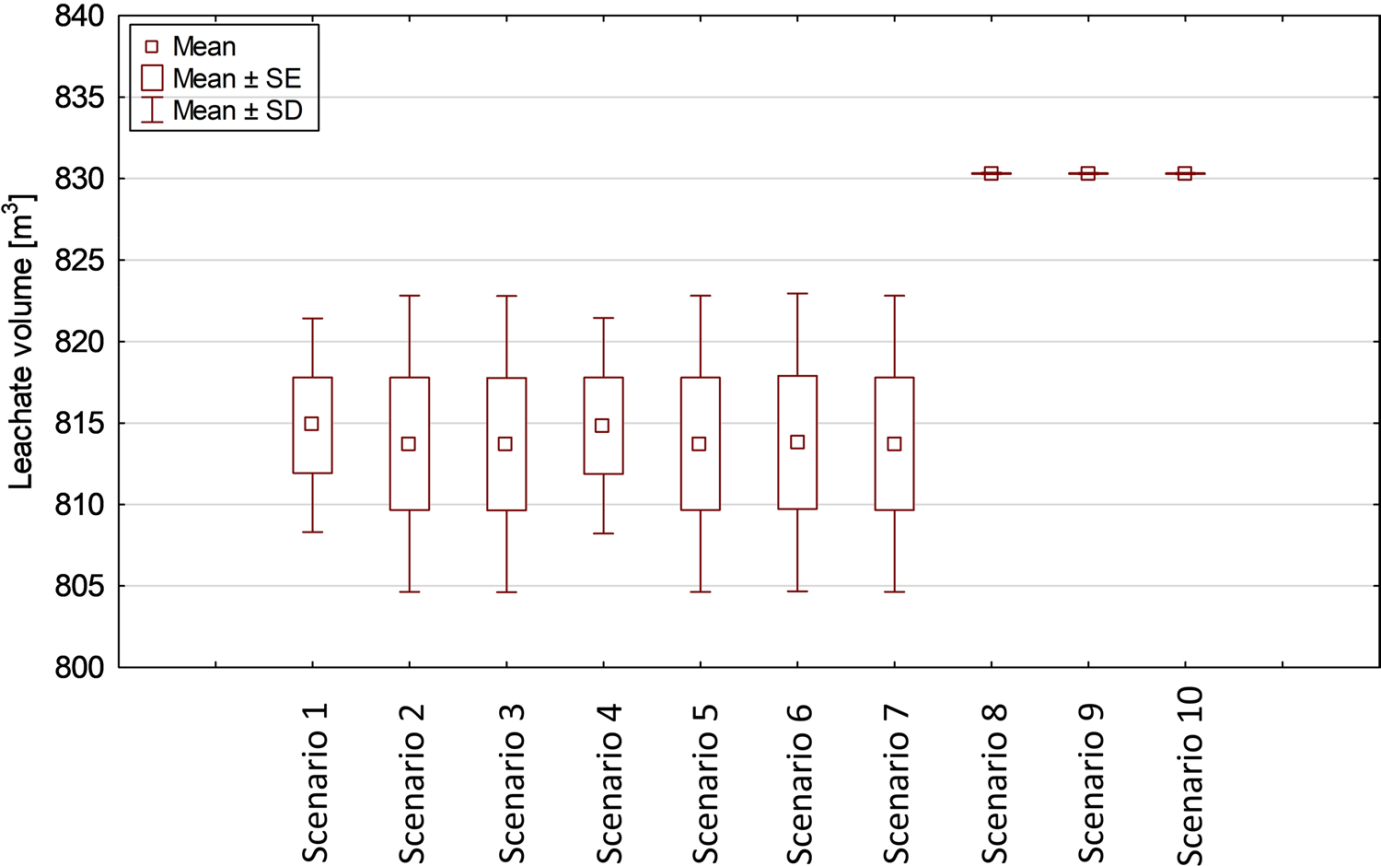
Components of water balance within the landfill site (leachate volume referred to 1 ha of the landfill)

Another factor which contributed to the higher leachate production rates could be the recirculation of the landfill leachate; however, recirculation was not analyzed in this study. It is also expected that leachate generation decreases over time due to stabilization of waste and reduced infiltration of rainwater through the landfill cover. For the analyzed Polish site conditions, it was found that the minimum amount of leachate generated (797.5 m^3^/year) occurs for scenario 3, in which the best placement quality (=5) is considered for PVC installed in the bottom of the landfill. The maximum leachate generation was revealed in scenarios 8 and 9 (830.4 m^3^/year) for which only three layers of bottom sealing were defined, and the worst placement quality (=1) was adopted for HDPE and LDPE, respectively. Significant differences in the amount of the leachate generated were found between scenarios 1–7 and 8–10. It was visible that in the case of the application of multilayer sealing systems, the leachate is generated in smaller amounts (difference of 33 m^3^/year between 10-layer and 7-layer systems) (Fig. 4).

**Fig. 5:**
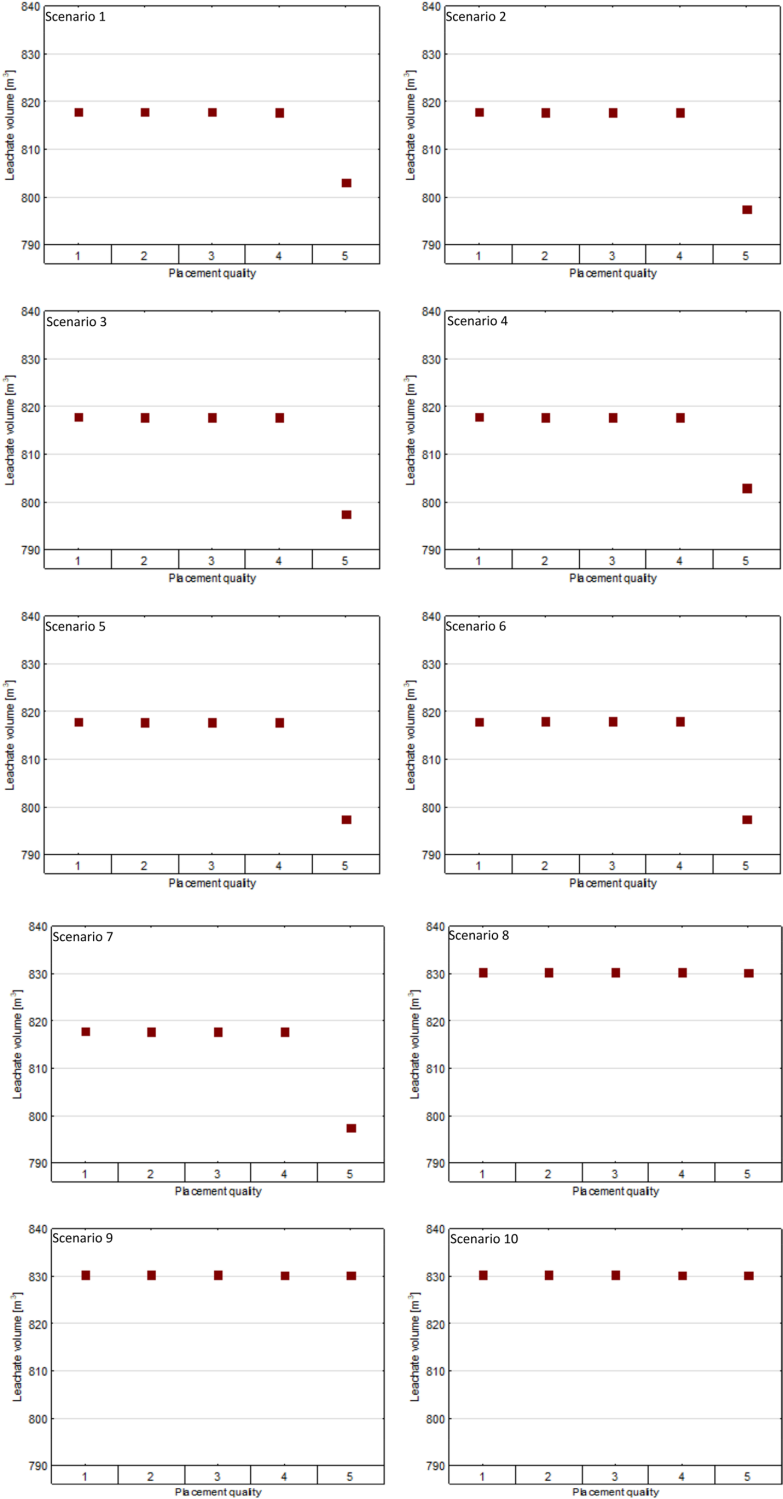
Impact of the placement quality of geomembrane liners on leachate generation (volume referred to 1 ha of the landfill)

When assessing the impact of placement quality of geomembrane liners on leachate generation, it was observed that in the scenarios 1–7 the leachate is generated in almost the same range (817.8 m^3^/year) when the placement quality is between 1 and 4. For the best placement quality (=5), there is a significant decrease in the leachate collected (from 14 to 20 m^3^/year), regarding scenarios 1–7. For scenarios 8–10 it was found that the amount of leachate collected (around 830 m^3^/year) is almost identical in each case, with no visible impact attributed to the placement quality of geomembrane liners (Fig. 5).

In scenario 1 in which the HDPE geomembrane is applied in the bottom sealing system, the quantity of leachate generated in the landfill ranges between 803 and 818 m^3^ per year from 1 ha of the landfill area, depending on the placement quality of the HDPE liner. Similar considerations can be attributed to the scenarios in which LDPE, PVC, BR, CPE, CSPE, and EPDM liners were applied.

For the placement quality in the range of 1–4, the leachate generation was close to 818 m^3^ per year from 1 ha of the landfill area. For the best placement quality (=5) of the geomembrane liner, it was revealed that in the scenarios in which LDPE, PVC, CPE, CSPE, and EPDM are applied, the amount of leachate generated per year from 1 ha of the landfill area was predicted around 797 m^3^. For cases with the best placement quality (=5) of HDPE and BR liners, the predicted value of leachate generated was 803 m^3^ per year from 1 ha of the landfill area. In the modeled scenarios with the application of three-layer bottom sealing systems, it was indicated that the volume of leachate generated did not range significantly with regard to the placement quality of the geomembranes used. For the last three of the analyzed cases, it was calculated that the leachate generated is close to 830 m^3^ per year from 1 ha of the landfill area. Depending on the type of geomembrane and its placement quality, it was proved that there are visible differences in the predicted percolation of leachate through the lowest soil layer applied in the bottom sealing system (Table 6).

**Table 6:** Rates of leachate percolation through the lowest soil layer depending on the type of geomembrane applied in the sealing system

Geomembrane material	Placement quality (-)				
	1	2	3	4	5
	Percolation through the lowest layer (m^3^/year)				
HDPE	3.811	0.030	0.030	0.030	0.001
LDPE	3.811	0.054	0.054	0.053	0.001
PVC	8.314	0.066	0.066	0.066	0.018
BR	8.307	0.030	0.030	0.030	0.002
CPE	8.309	0.056	0.056	0.056	0.006
CSPE	8.308	0.055	0.055	0.055	0.005
EPDM	8.308	0.055	0.055	0.055	0.004

It was found that in the case of the best placement quality (=5) of the geomembranes, the volume of percolating leachate is in the range 0.001–0.018 m^3^/year. The lowest percolation of leachate through the geomembrane is observed for HDPE and LDPE geomembranes, which can be also linked to the lowest hydraulic conductivity (10^−15^ m/s). In case of the PVC geomembrane, the highest possible percolation of the leachate was calculated, which can also be explained by the highest hydraulic conductivity (10^−13^ m/s) attributed to this material. The results indicate that reducing the hydraulic conductivity of the material by 1 order of magnitude can reduce the possible percolation of leachate even by up to two times. The results of the performed study have also pointed out that there are significant correlations between the parameters of the water budget balance in the landfill site (Table 7 and Table 8).

**Table 7:** Correlation matrix between the parameters of water balance for scenarios 1–5

Parameter	Precipitation	Runoff	Evapotranspiration	Change in water storage	Soil water	Snow water	Drainage
Scenario 1							
Precipitation	1.00000^a^	0.002250^e^	0.85721^b^	−0.03907^e^	0.23596^e^	0.40914^d^	0.66927^c^
Runoff	0.00225^e^	1.00000^a^	−0.42977^d^	−0.30914^d^	−0.53493^c^	−0.08901^e^	−0.17231^e^
Evapotranspiration	0.85721^b^	−0.42977^d^	1.00000^a^	0.00078^e^	0.52795^c^	0.28999^e^	0.56554^c^
Change in water storage	−0.03907^e^	−0.30914^d^	0.00078^e^	1.00000^a^	−0.61343^c^	0.87447^b^	−0.44893^d^
Soil water	0.23596^e^	−0.53493^c^	0.52795^c^	−0.61343^c^	1.00000^a^	−0.59136^c^	0.63722^c^
Snow water	0.40914^d^	−0.08901^e^	0.28999^e^	0.87447^b^	−0.59136^c^	1.00000^a^	−0.15613^e^
Drainage	0.66927^c^	−0.17231^e^	0.56554^c^	−0.44893^d^	0.63722^c^	−0.15613^e^	1.00000^a^
Scenario 2							
Precipitation	1.00000^a^	0.00225^e^	0.85721^b^	0.42393^d^	0.27687^e^	0.40914^d^	0.69811^c^
Runoff	0.00225^e^	1.00000^a^	−0.42977^d^	−0.73514^b^	−0.54661^c^	−0.08901^e^	−0.14725^e^
Evapotranspiration	0.85721^b^	−0.42977^d^	1.00000^a^	0.80562^b^	0.55643^c^	0.28999^e^	0.58292^c^
Change in water storage	0.42394^d^	−0.73514^c^	0.80562^b^	1.00000^a^	0.44125^d^	0.32179^d^	0.09631^e^
Soil water	0.27687^e^	−0.54661^c^	0.55643^c^	0.44125^d^	1.00000^a^	−0.55459^c^	0.65987^c^
Snow water	0.40914^d^	−0.08901^e^	0.28999^e^	0.32179^d^	−0.55459^c^	1.00000^a^	−0.13533^e^
Drainage	0.69812^c^	−0.14725^e^	0.58292^c^	0.09631^e^	0.65987^c^	−0.13533^e^	1.00000^a^
Scenario 3							
Precipitation	1.00000^a^	0.00225^e^	0.85721^b^	−0.08389^e^	0.22178^e^	0.40914^d^	0.69815^c^
Runoff	0.00225^e^	1.00000^a^	−0.42977^d^	−0.34923^d^	−0.58906^c^	−0.08901^e^	−0.14722^e^
Evapotranspiration	0.85721^b^	−0.42977^d^	1.00000^a^	−0.02601^e^	0.52513^c^	0.28999^e^	0.58293^c^
Change in water storage	−0.08389^e^	−0.34923^d^	−0.02601^e^	1.00000^a^	−0.53731^c^	0.84452^b^	−0.44814^d^
Soil water	0.22178^e^	−0.58906^c^	0.52513^c^	−0.53731^c^	1.00000^a^	−0.56129^c^	0.62310^c^
Snow water	0.40914^d^	−0.08901^e^	0.28999^e^	0.84452^b^	−0.56129^c^	1.00000^a^	−0.13526^e^
Drainage	0.69816^c^	−0.14722^e^	0.58293^c^	−0.44814^d^	0.62310^c^	−0.13526^e^	1.00000^a^
Scenario 4							
Precipitation	1.00000^a^	0.05809^d^	0.84934^b^	−0.88371^b^	0.56124^c^	0.09691^e^	0.79441^b^
Runoff	0.05809^d^	1.00000^a^	−0.41433^d^	−0.46444^d^	−0.78120^b^	0.28622^e^	−0.15764^e^
Evapotranspiration	0.84935^b^	−0.41433^d^	1.00000^a^	−0.50354^c^	0.81942^b^	0.19120^e^	0.62609^c^
Change in water storage	−0.88371^b^	−0.46444^d^	−0.50354^c^	1.00000^a^	−0.18993^e^	0.00682^e^	−0.74264^b^
Soil water	0.56124^c^	−0.78120^b^	0.81942^b^	−0.18993^e^	1.00000^a^	−0.31137^e^	0.69413^c^
Snow water	0.09691^e^	0.28622^e^	0.19120^e^	0.00682^e^	−0.31137^d^	1.00000^a^	−0.52656^c^
Drainage	0.79441^b^	−0.15764^e^	0.62609^c^	−0.74264^b^	0.69413^c^	−0.52656^c^	1.00000^a^
Scenario 5							
Precipitation	1.00000^a^	0.00225^e^	0.85721^b^	−0.03987^e^	0.55992^c^	0.40914^d^	0.69812^c^
Runoff	0.00225^e^	1.00000^a^	−0.42977^d^	−0.22986^e^	−0.24012^e^	−0.08901^e^	−0.14725^e^
Evapotranspiration	0.85721^b^	−0.42977^d^	1.00000^a^	−0.01986^e^	0.69090^c^	0.28999^e^	0.58292^c^
Change in water storage	−0.03988^e^	−0.22986^e^	−0.01986^e^	1.00000^a^	−0.72458^b^	0.88414^b^	−0.49937^d^
Soil water	0.55992^c^	−0.24012^e^	0.69090^c^	−0.72458^b^	1.00000^a^	−0.46538^d^	0.76401^b^
Snow water	0.40914^d^	−0.08901^e^	0.28999^e^	0.88414^b^	−0.46538^d^	1.00000^a^	−0.13532^e^
Drainage	0.69812^c^	−0.14725^e^	0.58292^c^	−0.49937^d^	0.76401^b^	−0.13532^e^	1.00000^a^

^a^Very high correlation

^b^High correlation

^c^Moderate correlation

^d^Low correlation

^e^Negligible correlation

**Table 8:** Correlation matrix between the parameters of water balance for scenarios 6–10

Parameter	Precipitation	Runoff	Evapotranspiration	Change in water storage	Soil water	Snow water	Drainage
Scenario 6							
Precipitation	1.00000^a^	0.00225^e^	0.85721^b^	−0.08381^e^	0.22178^e^	0.40914^d^	0.69814^c^
Runoff	0.00225^e^	1.00000^a^	−0.42977^d^	−0.34920^d^	−0.58906^c^	−0.08901^e^	−0.14724^e^
Evapotranspiration	0.85721^b^	−0.42977^d^	1.00000^a^	−0.02596^e^	0.52513^c^	0.28999^e^	0.58294^c^
Change in water storage	−0.08381^e^	−0.34920^d^	−0.02596^e^	1.00000^a^	−0.53733^c^	0.84456^b^	−0.44813^d^
Soil water	0.22178^e^	−0.58906^c^	0.52513^c^	−0.53733^c^	1.00000^a^	−0.56129^c^	0.62314^c^
Snow water	0.40914^d^	−0.08901^e^	0.28999^e^	0.84456^b^	−0.56129^c^	1.00000^a^	−0.13530^e^
Drainage	0.69814^c^	−0.14724^e^	0.58294^c^	−0.44813^d^	0.62314^c^	−0.13530^e^	1.00000^a^
Scenario 7							
Precipitation	1.00000^a^	0.00225^e^	0.85721^b^	−0.08381^e^	0.22178^e^	0.40914^d^	0.69812^c^
Runoff	0.00225^e^	1.00000^a^	−0.42977^d^	−0.34920^d^	−0.58906^c^	−0.08901^e^	−0.14726^e^
Evapotranspiration	0.85721^b^	−0.42977^d^	1.00000^a^	−0.02596^e^	0.52513^c^	0.28999^e^	0.58294^c^
Change in water storage	−0.08381^e^	−0.34920^d^	−0.02596^e^	1.00000^a^	−0.53733^c^	0.84457^b^	−0.44812^d^
Soil water	0.22178^e^	−0.58906^c^	0.52513^c^	−0.53733^c^	1.00000^a^	−0.56129^c^	0.62316^c^
Snow water	0.40914^d^	−0.08901^e^	0.28999^e^	0.84457^b^	−0.56129^c^	1.00000^a^	−0.13531^e^
Drainage	0.69812^c^	−0.14726^e^	0.58294^c^	−0.44812^d^	0.62316^c^	−0.13531^e^	1.00000^a^
Scenario 8							
Precipitation	1.00000^a^	0.00225^e^	0.85721^b^	−0.11463^e^	−0.21316^e^	0.40914^d^	0.70708^b^
Runoff	0.00225^e^	1.00000^a^	−0.42977^d^	−0.12933^e^	−0.74802^b^	−0.08901^e^	−0.25438^e^
Evapotranspiration	0.85721^b^	−0.42977^d^	1.00000^a^	−0.15402^e^	0.18394^e^	0.28999^e^	0.62241^c^
Change in water storage	−0.11464^e^	−0.12933^e^	−0.15402^e^	1.00000^a^	−0.49043^d^	0.85901^b^	−0.42615^d^
Soil water	−0.21316^e^	−0.74802^b^	0.18394^e^	−0.49043^d^	1.00000^a^	−0.58480^c^	0.33375^d^
Snow water	0.40914^d^	−0.08901^e^	0.28999^e^	0.85901^b^	−0.58480^c^	1.00000^a^	−0.04291^e^
Drainage	0.70709^b^	−0.25438^e^	0.62241^c^	−0.42615^d^	0.33375^d^	−0.04291^e^	1.00000^a^
Scenario 9							
Precipitation	1.00000^a^	0.00225^e^	0.85721^b^	−0.11499^e^	−0.53278^c^	0.40914^d^	0.70708^b^
Runoff	0.00225^e^	1.00000^a^	−0.42977^d^	−0.12894^e^	−0.79777^b^	−0.08901^e^	−0.25438^e^
Evapotranspiration	0.85721^b^	−0.42977^d^	1.00000^a^	−0.15484^e^	−0.09989^e^	0.28999^e^	0.62241^c^
Change in water storage	−0.11499^e^	−0.12894^e^	−0.15484^e^	1.00000^a^	−0.10854^e^	0.85882^b^	−0.42576^d^
Soil water	−0.53278^c^	−0.79777^b^	−0.09989^e^	−0.10854^e^	1.00000^a^	−0.39834^d^	−0.02831^e^
Snow water	0.40914^d^	−0.08901^e^	0.28999^e^	0.85882^b^	−0.39834^d^	1.00000^a^	−0.04291^e^
Drainage	0.70709^b^	−0.25438^e^	0.62241^c^	−0.42576^d^	−0.02831^e^	−0.04291^e^	1.00000^a^
Scenario 10							
Precipitation	1.00000^a^	−0.12502^e^	0.88578^b^	−0.29194^e^	0.16528^e^	0.40914^d^	0.72011^b^
Runoff	−0.12503^e^	1.00000^a^	−0.50392^c^	0.43404^d^	−0.49914^d^	−0.06556^e^	0.23995^e^
Evapotranspiration	0.88579^b^	−0.50392^c^	1.00000^a^	−0.62687^c^	0.54280^c^	0.15492^e^	0.53643^c^
Change in water storage	−0.29194^e^	0.43404^d^	−0.62687^c^	1.00000^a^	−0.97273^a^	0.65069^c^	−0.21247^e^
Soil water	0.16528^e^	−0.49914^d^	0.54280^c^	−0.97273^a^	1.00000^a^	−0.64513^c^	−0.01386^e^
Snow water	0.40914^d^	−0.06556^e^	0.15492^e^	0.65069^c^	−0.64513^c^	1.00000^a^	0.04176^e^
Drainage	0.72011^b^	0.23995^e^	0.53643^c^	−0.21247^e^	−0.01386^e^	0.04176^e^	1.00000^a^

^a^Very high correlation

^b^High correlation

^c^Moderate correlation

^d^Low correlation

^e^Negligible correlation

For each of the analyzed scenarios it was found that precipitation and evapotranspiration revealed very high or high positive correlation between each other. Depending on the scenario, the correlation between precipitation and the drainage of leachate was moderately or highly positive. This is in line with previous considerations which pointed out that in most scientific studies, precipitation is the main factor determining leachate generation in the landfill. In the current research, evapotranspiration has been also positively correlated with the drainage of leachate. Apart from rainfall, the leachate quantity may be also affected by evapotranspiration (Pazoki and Ghasemzadeh 2020). In each of the analyzed cases this correlation was found to be moderate. The leachate quantity is also influenced by the soil water. In all regarded scenarios, the impact of this factor on the drainage was moderately positive. There were also some observations made regarding the factors that have negligible impact on the leachate drainage. For the analyzed scenarios, the runoff has found to be such kind of factor. Moreover, snow water was described as a parameter with negligible correlation with drainage (with the exception of scenario 4).

The relationship between the leachate quantity and the amount of rainfall has been also studied in different regions of Poland. It was found by Talalaj (2015) that for municipal landfill in the north-eastern part of the country, the correlation between rainfall and the amount of leachate, expressed by the correlation coefficient, was equal to 0.48 (low correlation). Contrary to the above, a strong linear correlation between precipitation and leachate quantity (0.86) was revealed by Čeh et al. (2022) in the research performed on composting technology. Consequently, according to the findings of Abunama et al. (2021), it was proved that rates of leachate generation vary proportionally to the amount of precipitation. Likewise, Ehrig (1983) considered this component as the largest single contributor to the generation of landfill leachate and the most important factor for water input. The approach aiming at the estimation of the annual generation of leachate is found to be very useful when planning future landfill sites (Aziz et al. 2012). Important is also the fact that for climate zones where the annual precipitation is below 400 mm, all precipitation can be lost by evapotranspiration (Christensen et al. 1992). In this study, evapotranspiration accounted for about 71 to 82% of the annual precipitation. Following the results of the 5-year period of the analysis, the runoff accounted for almost 2 to 14% of the amount of precipitation. Other findings also showed that besides precipitation, a major part of the leachate generated comes from the water content in wastes (Frikha et al. 2017).

In the analyses related to the estimation of leachate generation in landfills, there is often a practice to express the leachate production as a percentage of annual precipitation. In this approach the amount of leachate generated is highly dependent on the technique of landfill operation and thus related to the use of specific machinery for waste compaction. Typical equipment to compact wastes includes compactors, dozers, and crawler tractors used under routine operations of the landfill (Owusu-Nimo et al. 2019; Ehrig 1983). It was revealed that the leachate generated in the landfill contributes to 14.8–17.3% of annual precipitation depending on the scenario. This is similar to the findings presented by Ehrig (1983) who stated that in a German landfill compacted with a steel wheel the leachate generated is equal to 15.1% of annual rainfall (652 mm). Ehrig (1983) also found that in a very young landfill the amount of leachate generated is in the range of 3.3–7.2% of annual rainfall (770 mm). When considering the compaction of wastes with the use of a crawler tractor, Ehrig (1983) observed that the percentage of rainfall that contributes to the generation of the leachate is visibly higher. The leachate generated was about 32.3% of annual rainfall (632 mm). Recirculation also enhances leachate generation. It was depicted that in the landfills operating with recirculation and with well-compacted wastes, the leachate generated may be even up to 38% of annual precipitation. Similar findings were reported for the Deir Al Balah landfill in the Gaza Strip (designed with a recirculation system), where the annual leachate contributed to 35.2% of total precipitation (Alslaibi et al. 2013). The research performed by Aziz et al. (2012) at the semi-aerobic landfill in Malaysia indicates that the leachate generated constitutes 9 to 14% of annual rainfall. For the site conditions in Greece, the reported value of leachate generated was 24.2% of the annual rainfall (Tatsi and Zouboulis 2002). In the study performed by Frikha et al. (2017) for the Tunisian site conditions, the leachate calculated using the HELP model was around 30–40% of precipitation. The important outcome of their study conducted in a semi-arid region was also the indication that the significant component of the water budget was evapotranspiration (77% of precipitation) and that in majority the leachate originated from the water content of the wastes. Similar conclusions were drawn by Alslaibi et al. (2013) who stated that half of the leachate discharged from the landfill comes from the moisture of the wastes disposed. Pantini et al. (2014) mentioned that besides rainfall, also water release is a key process of leachate generation, while evapotranspiration and runoff contribute to the leachate generation in 2% and 9%, respectively. Moreover, a significant impact on leachate percolation has the lining system which can cause the reduction of leachate percolation even up to 87%. Alslaibi et al. (2013) revealed that the absence of the recirculation system in the landfill can strengthen the reduction of leachate percolation up to 2.5%. In open landfills, where no upper cover layer was applied, the amount of leachate generated is proportionally correlated with precipitation. Depending on the actual meteorological conditions, the leachate generated can be up to 50–100% of the rainfall (Nas and Nas 2014).

## Conclusions

The presented research contributes to the broad field of waste management focused on the assessment of leachate generation for the purpose of proper landfill design and operation. The outcomes of the performed study indicate the possible range of leachate generation from MSW landfills, taking into account various practical solutions of cover and bottom sealing systems, with special attention given to the type of the geomembrane and its placement quality, as well as meteorological conditions of central Poland. It was proved by the performed study that the multilayer sealing system applied in the landfill may affect the reduction of leachate generation by up to 33 m^3^/year from 1 ha of the landfill. This reduction can be also influenced by the placement quality of the applied geomembrane, with the general conclusion that with the best placement quality (=5) of the material used, the lowest volumes of the leachate are expected. Nevertheless, this is true only for multilayer sealing systems, while when regarding three layers applied in the bottom sealing system, the impact of the placement quality of the geomembrane is not visible. The type and placement quality of the geomembrane should be also regarded in accordance with its hydraulic conductivity, which may significantly affect leachate percolation (even a two times reduction of percolating leachate observed with the reduction of the hydraulic conductivity by one order of magnitude). The adopted methodological approach and described outcomes, discussed with several examples of leachate amount calculations, supplement the existing knowledge on wastewater management, with particular attention given to the amount of leachate that can be generated in different site conditions and different technological solutions adopted for the cover and the bottom sealing systems.

## Data Availability

Data sharing is not applicable to this article.
